# J-CKD-DB: a nationwide multicentre electronic health record-based chronic kidney disease database in Japan

**DOI:** 10.1038/s41598-020-64123-z

**Published:** 2020-04-30

**Authors:** Naoki Nakagawa, Tadashi Sofue, Eiichiro Kanda, Hajime Nagasu, Kunihiro Matsushita, Masaomi Nangaku, Shoichi Maruyama, Takashi Wada, Yoshio Terada, Kunihiro Yamagata, Ichiei Narita, Motoko Yanagita, Hitoshi Sugiyama, Takashi Shigematsu, Takafumi Ito, Kouichi Tamura, Yoshitaka Isaka, Hirokazu Okada, Kazuhiko Tsuruya, Hitoshi Yokoyama, Naoki Nakashima, Hiromi Kataoka, Kazuhiko Ohe, Mihoko Okada, Naoki Kashihara

**Affiliations:** 1 Division of Cardiology, Nephrology, Respiratory and Neurology, Department of Internal Medicine, Asahikawa Medical University, Asahikawa, Japan; 2 Division of Nephrology and Dialysis, Department of Cardiorenal and Cerebrovascular Medicine, Kagawa University, Kagawa, Japan; 3 Medical Science, Kawasaki Medical School, Kurashiki, Japan; 4 Department of Nephrology and Hypertension, Kawasaki Medical School, Kurashiki, Japan; 5 Department of Epidemiology, Johns Hopkins Bloomberg School of Public Health, Baltimore, USA; 6 Division of Nephrology and Endocrinology, University of Tokyo Graduate School of Medicine, Tokyo, Japan; 7 Division of Nephrology, Nagoya University Graduate School of Medicine, Nagoya, Japan; 8 Division of Nephrology, Department of Nephrology and Laboratory Medicine, Kanazawa University, Kanazawa, Japan; 9 Department of Endocrinology, Metabolism and Nephrology, Kochi Medical School, Kochi University, Kochi, Japan; 10 Department of Nephrology, Faculty of Medicine, University of Tsukuba, Tsukuba, Japan; 11 Division of Clinical Nephrology and Rheumatology, Niigata University Graduate School of Medical and Dental Sciences, Niigata, Japan; 12 Department of Nephrology, Graduate School of Medicine, Kyoto University, Kyoto, Japan; 13 Department of Human Resource Development of Dialysis Therapy for Kidney Disease, Okayama University Graduate School of Medicine, Dentistry and Pharmaceutical Sciences, Okayama, Japan; 14 Division of Nephrology, Department of Internal Medicine, Wakayama Medical University, Wakayama, Japan; 15 Division of Nephrology, Faculty of Medicine, Shimane University, Izumo, Japan; 16 Department of Medical Science and Cardiorenal Medicine, Yokohama City University Graduate School of Medicine, Yokohama, Japan; 17 Department of Nephrology, Osaka University Graduate School of Medicine, Suita, Japan; 18 Department of Nephrology, Faculty of Medicine, Saitama Medical University, Saitama, Japan; 19 Department of Integrated Therapy for Chronic Kidney Disease, Kyushu University, Fukuoka, Japan; 20 Department of Nephrology, Nara Medical University, Kashihara, Japan; 21 Department of Nephrology, Kanazawa Medical University School of Medicine, Ishikawa, Japan; 22 Department of Advanced Information Technology, Kyushu University, Fukuoka, Japan; 23 Faculty of Health Science and Technology, Kawasaki University of Medical Welfare, Kurashiki, Japan; 24 Department of Healthcare Information Management, The University of Tokyo Hospital, Tokyo, Japan; 25 Institute of Health Data Infrastructure for All, Tokyo, Japan

**Keywords:** Chronic kidney disease, Epidemiology

## Abstract

The Japan Chronic Kidney Disease (CKD) Database (J-CKD-DB) is a large-scale, nation-wide registry based on electronic health record (EHR) data from participating university hospitals. Using a standardized exchangeable information storage, the J-CKD-DB succeeded to efficiently collect clinical data of CKD patients across hospitals despite their different EHR systems. CKD was defined as dipstick proteinuria ≥1+ and/or estimated glomerular filtration rate <60 mL/min/1.73 m^2^ base on both out- and inpatient laboratory data. As an initial analysis, we analyzed 39,121 CKD outpatients (median age was 71 years, 54.7% were men, median eGFR was 51.3 mL/min/1.73 m^2^) and observed that the number of patients with a CKD stage G1, G2, G3a, G3b, G4 and G5 were 1,001 (2.6%), 2,612 (6.7%), 23,333 (59.6%), 8,357 (21.4%), 2,710 (6.9%) and 1,108 (2.8%), respectively. According to the KDIGO risk classification, there were 30.1% and 25.5% of male and female patients with CKD at very high-risk, respectively. As the information from every clinical encounter from those participating hospitals will be continuously updated with an anonymized patient ID, the J-CKD-DB will be a dynamic registry of Japanese CKD patients by expanding and linking with other existing databases and a platform for a number of cross-sectional and prospective analyses to answer important clinical questions in CKD care.

## Introduction

Chronic kidney disease (CKD) is not only a precursor of end-stage renal disease (ESRD) but also a strong risk factor for various adverse outcomes such as cardiovascular disease (CVD) and dementia^1–3^. It is estimated that 10 to 12% (over 10 million people) of Japanese adults have CKD^4–6^, and there is a concern that the prevalence of CKD will be even higher in the future due to aging population. Therefore, it is urgently necessary to understand the patterns of CKD progression and investigate how to optimize preventive and therapeutic strategies through epidemiological and clinical research. To collect clinical data from CKD patients in Japan and address important clinical questions, the Committee for the Working Group for Renal Biopsy Database in the Japanese Society of Nephrology (JSN) established a nationwide, web-based, and prospective registry system in 2007. This led to the development of two registries — one with patients who underwent renal biopsy, called the Japan Renal Biopsy Registry (J-RBR)^7^, and the other with those who did not undergo renal biopsy, called the Japan Kidney Disease Registry (J-KDR)^8^. These registries have contributed to better understanding the epidemiology of biopsied and unbiopsied renal disease in Japan; however, these registries rely on manual online data entry and thus have a few important limitations with regard to scaling up (i.e., number of patients and types of variables) and data accuracy.

Recently, the application of Information and Communication Technology (ICT) to the medical field has established a foundation for big data analysis informing clinical management^9,10^. In medical institutions, enormous amount of electronic health record (EHR) data are accumulated daily^11,12^. However, there are several EHR systems that may not be able to communicate each other, precluding multicentre EHR-based research. In this context, the Ministry of Health, Labor and Welfare has developed a system, the Standardized Structured Medical Information eXchange (SS-MIX2)(http://www.ss-mix.org/consE/)^13^, which allows us to compile EHR data from different systems. Utilizing SS-MIX2, the JSN and the Japan Association for Medical Informatics have constructed a comprehensive clinical database of CKD patients called the Japan Chronic Kidney Disease Database (J-CKD-DB) through the collaboration with 21 university hospitals nationwide as of January 2018. The main aim of the J-CKD-DB registry is to develop a large-scale, nation-wide registry based on EHR data from the participating university hospitals to conduct investigations of the actual practice pattern of CKD in Japan, including cross-sectional and prospective studies. Here, we described the process of establishing J-CKD-DB, summarized characteristics of initial 39,121 CKD outpatients in J-CKD-DB, and discussed major research questions to be addressed in J-CKD-DB.

## Results

### Development of J-CKD-DB

**Figure 1:**
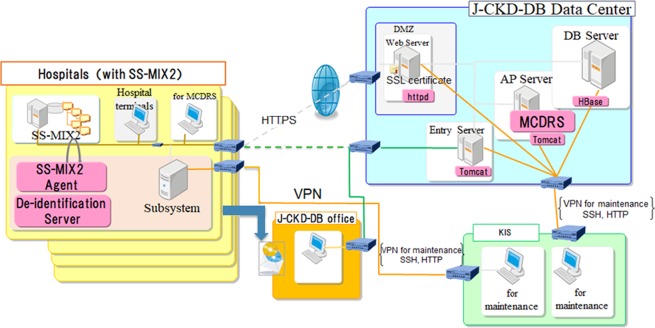
Overview of the Japan Chronic Kidney Disease Database (J-CKD-DB) System. The SS-MIX2 (Standardized Structured Medical Information eXchange) leveraged recent progress made in healthcare information standards in Japan, including code standardization regarding laboratory data items and prescription data. The university hospitals participating in J-CKD-DB (left boxes) needed to have electronic health record systems that incorporated SS-MIX2 storage and a template-based structured-data entry function that could transfer the entered data to the SS-MIX2 storage. All data elements are extracted semi-automatically using SS-MIX2 storage and send to J-CKD-DB data centre through HTTPS (upper right box). MCDRS (Multi-purpose Clinical Data Repository System), a software system developed at the University of Tokyo, is adopted for designing and collecting the data elements. The administrative office of J-CKD-DB project is in Kawasaki Medical School (lower middle box). J-CKD-DB is maintained, and data cleaning is carried out at the office (lower right box). AP, application; DB, database; DMZ, demilitarized zone; HTTPS, hypertext transfer protocol secure; SSH, secure shell; SSL, secure sockets layer; VPN, virtual private network.

Figure 1 shows the overview of the J-CKD-DB system. The J-CKD-DB is a nationwide multicentre EHR-based database of CKD in Japan. The inclusion criteria of the J-CKD-DB were as follows: (1) age ≥ 18 years old and (2) proteinuria ≥ 1 + (dipstick test) and/or estimated glomerular filtration rate (eGFR) <60 mL/min/1.73 m^2^. All patients meeting these inclusion criteria based on outpatient or inpatient information were registered in the J-CKD-DB regardless of whether patients were under the care of nephrology or other specialties.

Among more than 80 university hospitals in Japan, 21 agreed to join the J-CKD-DB Registry (Supplementary Table S1). Table 1 summarizes information collected from the J-CKD-DB. The Multipurpose Clinical Data Registry System (MCDRS) for data extraction and registry has been developed through the Funding Program for World-Leading Innovative R&D on Science and Technology (FIRST Program) by the Japan Society for Promotion of Science; this system allows the efficient collection of clinical data using the SS-MIX2 format^11^, which is application-independent, and current use includes community healthcare information systems, backup for disaster, multi-institutional database development, and clinical research.

**Table 1:** Measures assessed at the baseline and during follow-up in the J-CKD-DB.

	Data elements		Description/unit
1	Hospital code	Issued by J-CKD-DB administration office	4 digit code
2	Birth year & month		YYYYMM
3	Gender		M: male, F: female
4	Clinical department		3 digit code
5	Exceptional cases		1: haemodialysis, 2: peritoneal dialysis, 3: renal transplantation, 99: others
6	Start date of treatment		YYYYMMDD
7	Patient		I: inpatient, O: outpatient
8	Outcome	Repeat as necessary	01: discharged, 05: referred to other hospitals, 20: deceased
9	Date of admission	Repeat as necessary	YYYYMMDD
10	Date of discharge	Repeat as necessary	YYYYMMDD
11	Date of laboratory test	Repeat as necessary	YYYYMMDD
12	Serum creatinine level	Repeat as necessary -Laboratory test code (JLAC10 code) -Laboratory test value -Laboratory test unit -Date -Inpatient/outpatient	mg/dL
13	Protein urine (qualitative)		-, ±, +, 2 + , 3 + , 4+
14	Urinary occult blood (qualitative)		-, ±, +, 2 + , 3 + , gross
15	urine protein-to-creatinine ratio		g/gCre
16	Protein urine (per day)		g/day
17	Protein urine (quantitative)		mg/dL
18	Urine protein-to-creatinine ratio		mg/gCre
19	Urinary albumin (per day)		mg/day
20	Urine creatinine (random urine)		mg/dL
21	Urine creatinine (pooled urine)		mg/dL
22	Urinary sodium (random urine)		mEq/L
23	Urinary sodium (pooled urine)		mEq/L
24	Urea nitrogen (random urine)		mg/dL
25	Urea nitrogen (pooled urine)		mg/dL
26	Urine Volume		mL/day
27	Total serum protein		g/dL
28	Serum albumin		g/dL
29	Uric acid		mg/dL
30	Urea-nitrogen		mg/dL
31	Serum sodium	Repeat as necessary -Laboratory test code (JLAC10 code) -laboratory test value -laboratory test unit -date -inpatient/outpatient	mEq/L
32	Serum potassium		mEq/L
33	Serum chloride		mEq/L
34	Serum magnesium		mg/dL
35	Total cholesterol		mg/dL
36	HDL cholesterol		mg/dL
37	LDL cholesterol		mg/dL
38	Triglyceride		mg/dL
39	HbA1c		%
40	Glycated albumin		%
41	Cystatin C		mg/L
42	Serum calcium		mg/dL
43	Serum phosphate		mg/dL
44	Intact PTH		pg/mL
45	Whole PTH		pg/mL
46	Serum iron		μg/dL
47	TIBC		μg/dL
48	TSAT		%
49	Ferritin		ng/mL
50	Bicarbonate concentration		mEq/L
51	BNP		pg/mL
52	CRP		mg/dL
53	WBC		/μL
54	RBC		/μL
55	Haemoglobin		g/dL
56	Haematocrit		%
57	PLT		/μL
58	AST		U/L
59	ALT		U/L
60	Antinuclear antibody-FA		times
61	Antinuclear antibody-EIA		
62	MPO-ANCA(EIA)		U/mL
63	MPO-ANCA (CLEIA)		times
64	PR3-ANCA (EIA)		U/mL
65	PR3-ANCA (CLEIA)		U/mL
66	anti-GBM antibody (EIA)		U/mL
67	anti-GBM antibody (CLEIA)		U/mL
68	serum complement titer (CH50)		U/mL
69	Prescriptions	Repeat as necessary -Date -Pharmaceutical product code -Dosage -Rout of administration -Duration of administration	Repeat for each pharmaceutical product
70	Diagnosis	Use ICD10 compliant Standard Master	

HDL, high-density lipoprotein; LDL, low-density lipoprotein; HbA1c, glycated haemoglobin; PTH, parathyroid hormone; TIBC, Total iron binding capacity; TSAT, Transferrin saturation; BNP, Brain natriuretic peptide; CRP, C-reactive protein; WBC, white blood cell count; RBC, red blood cell count; PLT, platelet count; AST, aspartate aminotransferase; MPO-ANCA, myeloperoxidase-antineutrophil cytoplasmic antibody; PR3-ANCA, Proteinase 3 antineutrophil cytoplasmic antibody; GBM, glomerular basement membrane; JLAC10, Japan Laboratory Code Version 10; ICD10, International Classifications of Disease, 10th revision.

### Participant selection and baseline characteristics

Supplementary Fig. 1 shows the selection flowchart for an initial analysis which is focused on CKD outpatients. As of January 2018, over 100,000 CKD patients from 11 university hospitals (Phase 1 Database-Building hospitals) were registered in the database from 1 January 2014 to 31 December 2014. Four university hospitals had no information about admission history as of January 2018 and thus were not included. Finally, 39,121 outpatients without admission history were included from 7 university hospitals in the initial analysis.

The key findings of the initial analysis are shown in Table 2. Among the 39,121 CKD outpatients (median age was 71 years, 54.7% were men, median eGFR was 51.3 mL/min/1.73 m^2^), we observed that the numbers of patients with CKD stage G1 (eGFR ≥ 90 mL/min/1.73 m^2^), G2 (60–89), G3a (45–59), G3b (30–44), G4 (15–30), and G5 ( < 15) were 1,001 (2.6%), 2,612 (6.7%), 23,333 (59.6%), 8,357 (21.4%), 2,710 (6.9%), and 1,108 (2.8%), respectively (Table 2), according to the CKD stages in the KDIGO guideline^9^. Among the 19,055 cases (48.7%) with available proteinuria data, the number of patients with CKD stage A1 (negative proteinuria), A2 (trace proteinuria [±]), and A3 (dipstick proteinuria ≥1+) were 9,357 (49.1%), 3,126 (16.4%), and 6,572 (34.5%), respectively (Table 2).

**Table 2:** General characteristics of CKD outpatients in the J-CKD-DB at baseline.

*N*	39,121
Age [IQI] (years)	71 [62–79]
Age category	
18–45 years	2,424 (6.2%)
45–64 years	9,166 (23.4%)
65–74 years	12,303 (31.4%)
75–84 years	11,695 (9.9%)
≥85 years	3,533 (9.0%)
Gender: Male	21,410 (54.7%)
eGFR [IQI] (mL/min/1.73 m^2^)	51.3 [42.2–57.0]
eGFR stage	
G1	1,001 (2.6%)
G2	2,612 (6.7%)
G3a	23,333 (59.6%)
G3b	8,357 (21.4%)
G4	2,710 (6.9%)
G5	1,108 (2.8%)
Dipstick proteinuria	Overall: 19,055
(−)	9,357 (49.1%)
(±)	3,126 (16.4%)
(1+)	3,783 (19.9%)
(2+)	1,919 (10.1%)
(3+)	784 (4.1%)
(4+)	86 (0.5%)
Haemoglobin (SD) (g/dL)	13.2 (1.9)
Serum albumin (SD) (g/dL)	4.1 (0.5)
Serum uric acid (SD) (mg/dL)	5.9 (1.5)
Serum total cholesterol (mg/dL)	186.4 (38.6)
Serum sodium (SD) (mEq/L)	141.4 (2.8)
Serum potassium (SD) (mEq/L)	4.3 (0.5)
Serum chloride (SD) (mEq/L)	104.5 (3.3)
Serum calcium (SD) (mg/dL)	9.1 (0.5)
Serum phosphate (SD) (mg/dL)	3.4 (0.9)
Serum C-reactive protein [IQI] (mg/dL)	0.10 [0.04–0.30]

Continuous variables are described as median [inter-quartile interval, (IQI)] or mean (standard deviation, SD). Factors are described as n (%).

### Clinical features of CKD outpatients in japan

**Figure 2:**
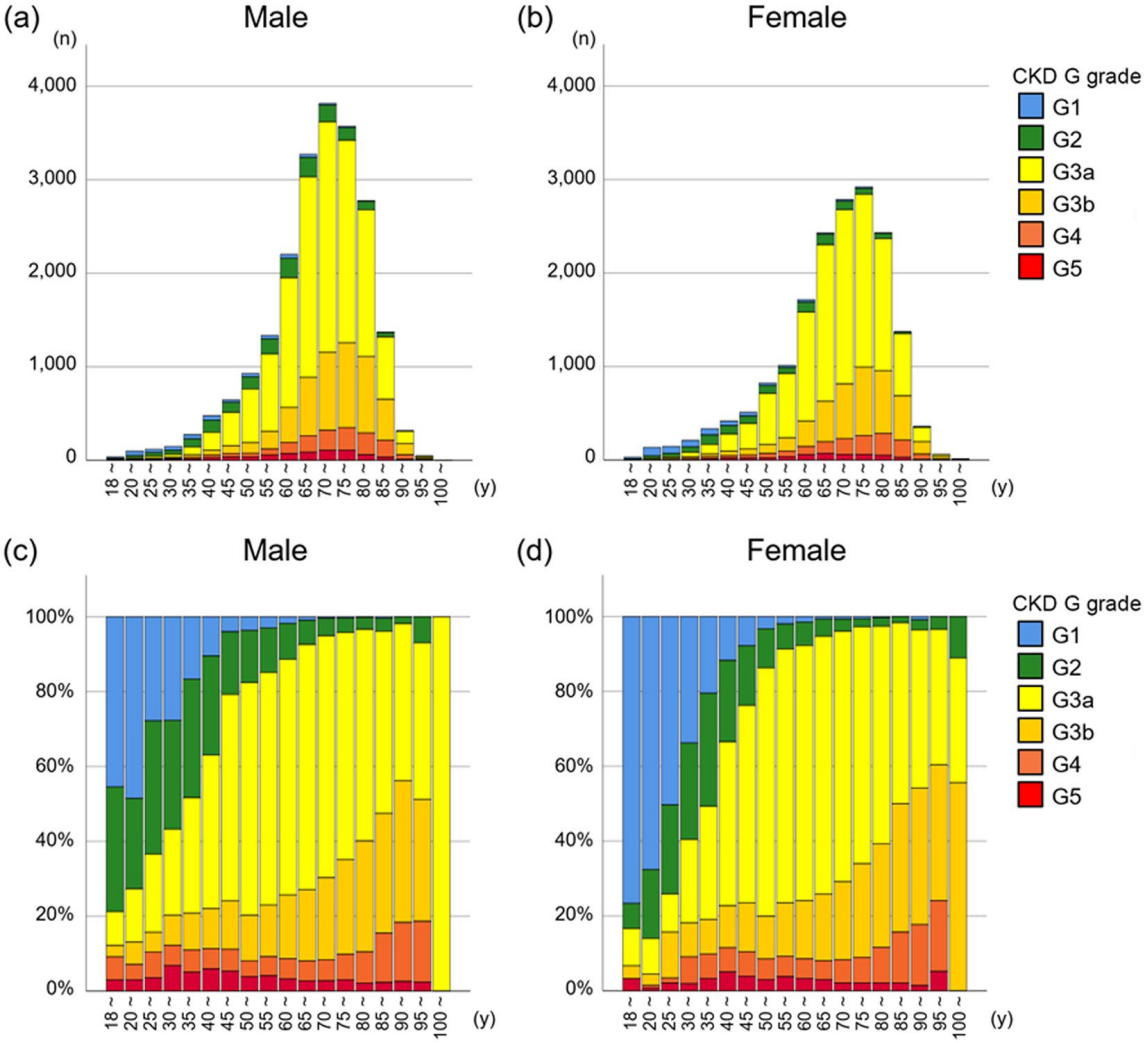
Age distribution (**a**,**b**) and proportion (**c**,**d**) of CKD G stage by sex in the J-CKD-DB according to the KDIGO criteria.

**Figure 3:**
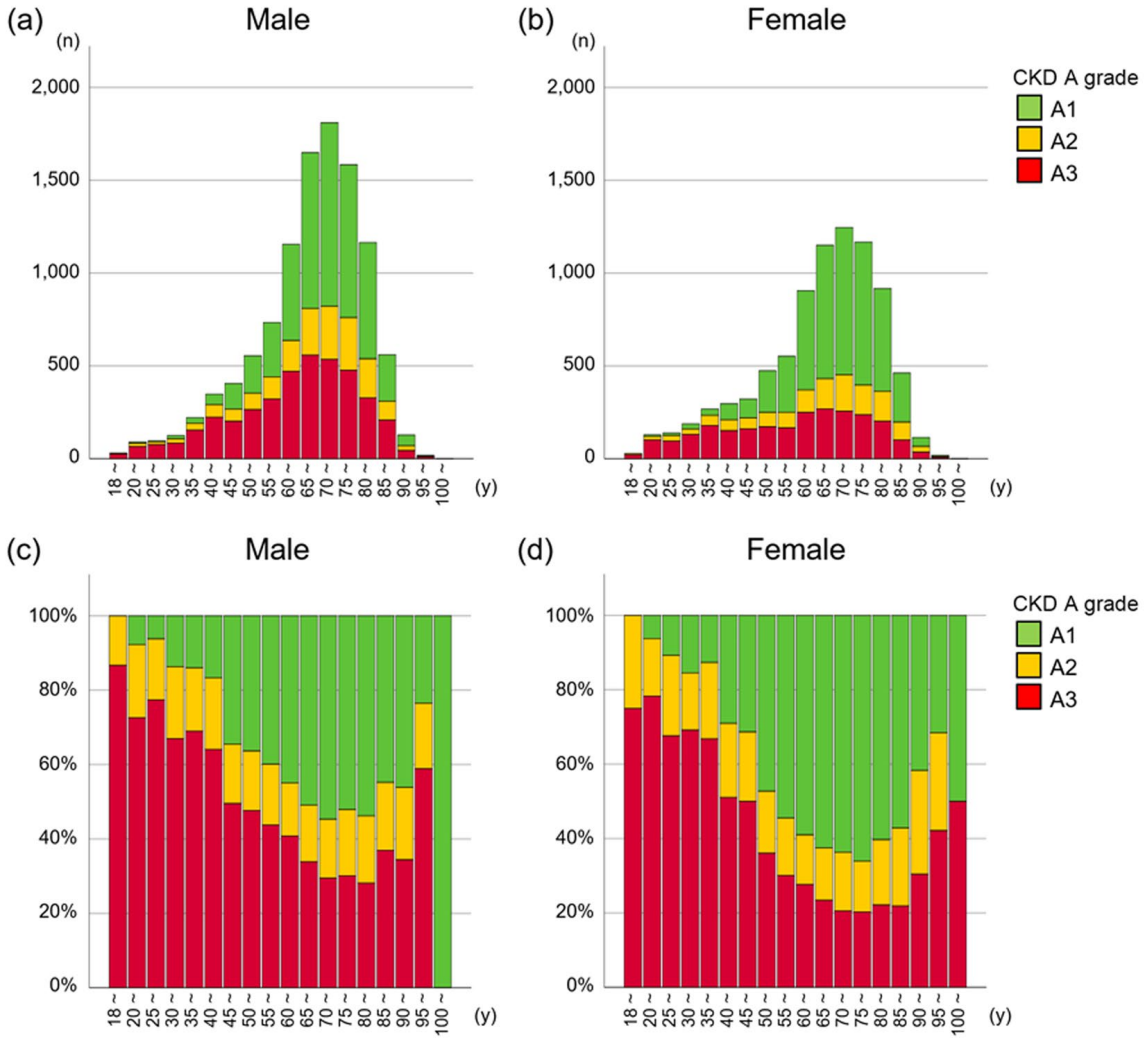
Age distribution (**a**,**b**) and proportion (**c**,**d**) of CKD A stage by sex in the J-CKD-DB according to the KDIGO criteria.

As anticipated, majority of CKD patients were older than 65 years old, with the most prevalent age category of 70–79 years in both sexes (Fig. 2a,b). The most prevalent G category after the age of 40 was G3 in both sexes. The proportion of G3b and G4 increased gradually along with age in both sexes (Fig. 2c,d, Supplementary Table S2). The proportion of CKD stage G5 was higher in younger patients (Fig. 2c,d, Supplementary Table S2). In all age groups, proteinuria was more predominant in men than in women (Fig. 3a,b, Supplementary Table S3). The proportion of those with CKD stage A3 was higher among younger patients (60%–80%) than among older ones (20%–40%) (Fig. 3c,d), suggesting that younger patients with advanced CKD are more likely to be referred to and managed in university hospitals than are older patients.

**Figure 4:**
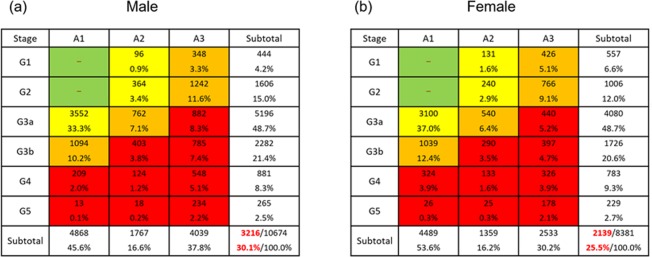
The KDIGO risk classification by age and sex in the J-CKD-DB. According to the KDIGO risk classification, very high-risk (red zone) cases accounted for 30.1% and 25.5% of all cases of male (**a**) and female (**b**) patients, respectively.

**Figure 5:**
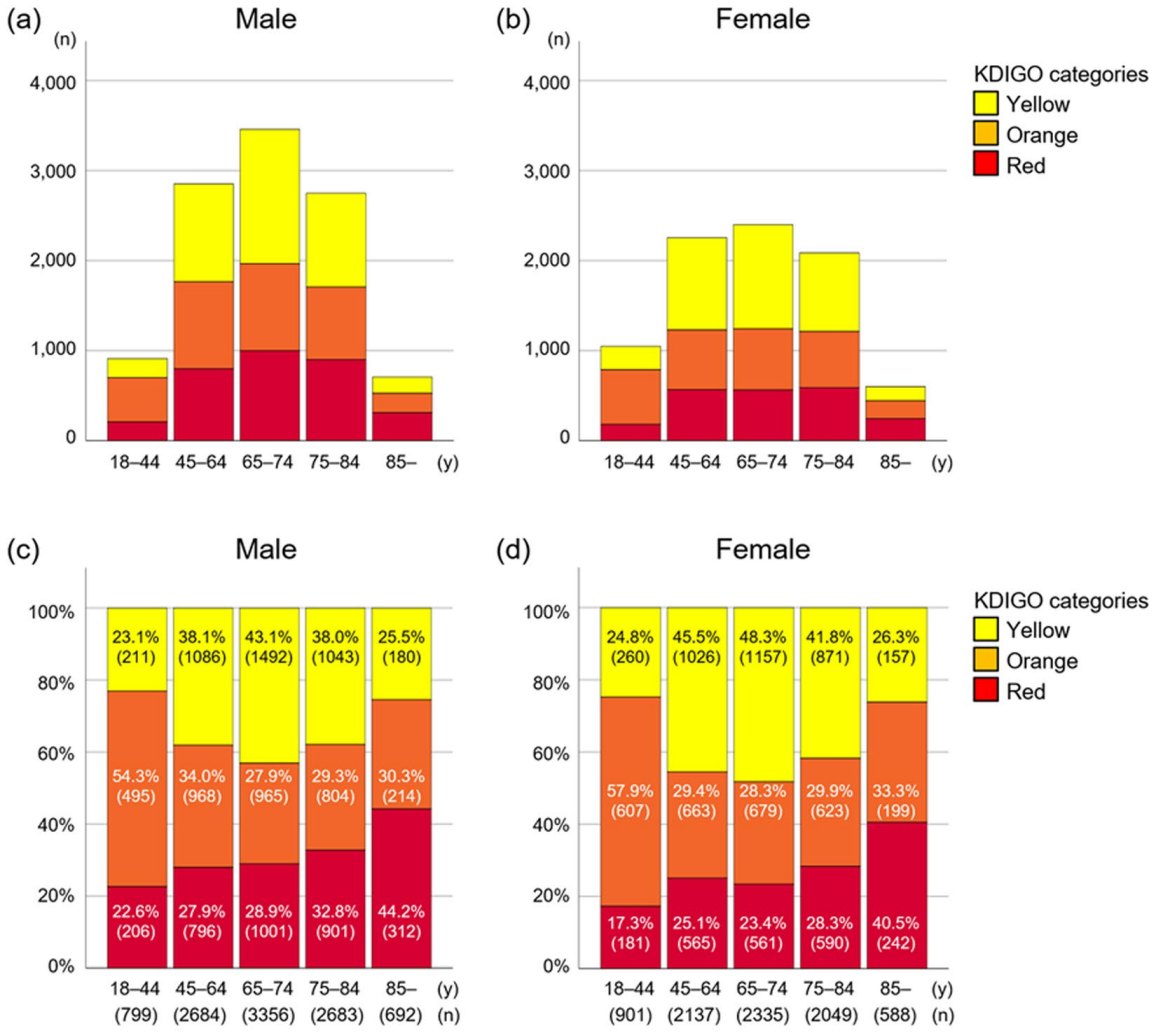
Age distribution (**a**,**b**) and proportion (**c**,**d**) of patients by KDIGO risk categories in the J-CKD-DB. The highest proportion of very high-risk (red zone) cases in both sexes is among patients over 85 years of age (**c**,**d**).

According to the KDIGO risk classification, very high-risk (red zone in Fig. 4a,b) cases accounted for 30.1% and 25.5% of all cases of male and female patients, respectively. Through all age strata, the very high-risk (red zone) cases were higher in males than in female (Fig. 5a,b). Among young patients 18–44 years, the most prevalence risk category was high-risk (orange zone), accounting for 54.3% in male and 57.9% in female (Fig. 5c,d). The prevalence of very high-risk (red zone) CKD increased gradually along with age in both sexes (i.e., 44.2% in male and 40.5% in female among patients over 85 years) (Fig. 5c,d).

## Discussion

In the present study, we have established the largest registry of CKD patients in Japan, called the J-CKD-DB based on advanced EHR systems to automatically extract data. As an initial analysis, we analyzed 39,121 CKD outpatients and observed that majority of CKD patients were older than 65 years old, with the most prevalent age category of 70–79 years in both sexes. Younger CKD patients in J-CKD-DB tended to be at more advanced A stages than older patients.

**Figure 6:**
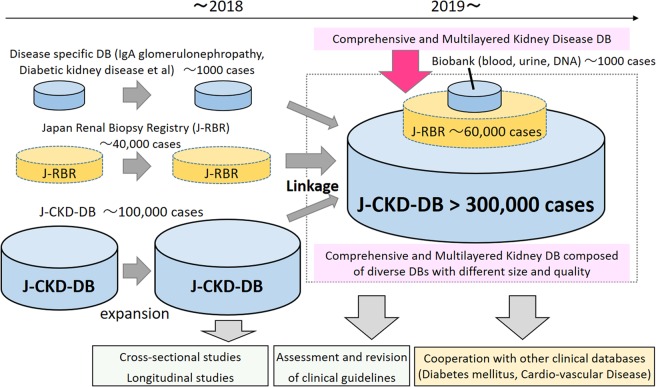
The plan for the J-CKD-DB project. The J-CKD-DB (bottom layer) will be integrated into other databases including J-RBR/J-KDR (Japan Renal Biopsy Registry and Japan Kidney Disease Registry) (middle layer). It is also planning to connect J-CKD-DB to biological samples and genomic information after informed consent (top layer) and establishing a multi-layered database that will comprise the J-RBR. Numbers are number of patients/measurements.

A major strength of the J-CKD-DB is the largest registry of CKD patients in Japan. Since the all data elements are extracted automatically using SS-MIX2 storage as determined from EHR data^11,12^, data collection and analysis will be facilitated by the J-CKD-DB in several ways. First, it is possible to build a multi-layered database by linking with an existing database, such as J-RBR/J-KDR^7,8^ for complementary purposes (Fig. 6). Furthermore, we are also planning to connect the J-CKD-DB to biological samples (blood, serum and urine) and genomic information after informed consent and establish a multi-layered database that will comprise the J-RBR/J-KDR at the middle layer and a biological sample database at the top layer and the J-CKD-DB at the bottom layer (Fig. 6). Although there are some systems to build up the database of CKD automatically in the world^14–17^, the J-CKD-DB has an advantage of expanding and linking with these multi-layer databases over the other systems. Second, it is possible to conduct various studies by publicly inviting research questions, which will provide evidence on CKD management. Third, the database can be used to analyse guideline-based quality indicators, compliance rates, and the heterogeneity of medical quality across institutions. By iterating the above processes, the J-CKD-DB can develop evidence-based strategies, informing future clinical guidelines and improving the quality of medical treatment for CKD patients.

In the process of establishing the J-CKD-DB, we noticed a few issues to be addressed. Although laboratory and prescription data have generally been described according to the standardized code system, SS-MIX2, throughout Japan, we found variations in those coding systems across the participating hospitals^11^. We found it challenging to convert the local code of medical care and tests to the standardized code by referencing mapping tables. One solution is to consider local variations, which will contribute to the construction of additional clinical effect databases in the future.

The present study demonstrated that clinical features of 39,121 CKD outpatients in university hospitals in Japan. As anticipated, the prevalence of proteinuria increased as GFR decreased. Male patients in J-CKD-DB tended to have higher levels of proteinuria than female patients. Accordingly, the prevalence of CKD with very high-risk (according to the KDIGO risk classification) was higher in male than in female patients across all age strata. When we looked the proportion of CKD risk stages within each age category, the highest proportion of high-risk (orange zone) CKD was seen among younger patients aged 18–44 years, with high proportion of CKD stage G5 and A3 in both sexes. In the field of adolescent and young adult patients with childhood-onset CKD, progressive CKD due to various diseases including childhood-onset nephrotic syndrome, chronic glomerulonephritis such as IgA nephropathy, congenital malformations of the kidney and urinary tract (CAKUT), persist after patients become adults^18^. Especially, the median age at which CAKUT progresses to ESKD was reported to be around 35 years^19^. Based on these reports, younger patients with advanced CKD are more likely to be referred to and managed in university hospitals. Overall, the prevalence of proteinuria was higher in more severe stages of eGFR, although only 48.7% of CKD patients in J-CKD-DB had data on proteinuria. Further, we will present the results of cross-sectional analyses in more detail and collecting more patients to conduct longitudinal follow-up study for patients in the J-CKD-DB as a J-CKD-DB extension (J-CKD-DB-Ex).

Weaknesses of the J-CKD-DB are as follows: First, EHR data may be inferior to observational epidemiological studies in some information, e.g. the cause of CKD, body mass index, blood pressure levels, social status, patient-reported experience measures, and incidence of CVDs because these elements are not included in SS-MIX2. Hence, some research questions related to these variables were not answered. Second, eGFR values based on a single measurement of serum creatinine are prone to causing misclassification, specifically in CKD G3a without proteinuria, thus not meeting the chronicity criterion. However, this approach has been used often in CKD research^20,21^.

In conclusion, we launched the J-CKD-DB based on advanced EHR systems to automatically extract data literally and literally eliminate manual input. As the information from every clinical encounter will be updated with an anonymized patient ID, the J-CKD-DB will be used for both cross-sectional and prospective investigations for patients with CKD in Japan to answer important clinical research questions and contribute to the improvement in the quality of CKD care.

## Materials and Methods

### Study design

This was an observational retrospective study using electronic health records.

### Setting and data source

The J-CKD-DB has been designed as a nationwide multicentre EHR-based database of patients with CKD in collaboration with the JSN and the JAMI. It was initiated in June 2016 as a comprehensive database of the clinical effective information of the Ministry of Health, Labor and Welfare (UMIN trial number, UMIN000026272). To ensure the smooth implementation and maintenance of the systems essential for J-CKD-DB; the facilities participating in J-CKD-DB needed to have EHR systems that incorporated SS-MIX2 storage (Fig. 1)^11^. The ethical committee of Kawasaki Medical School and JSN comprehensively approved the study (JSN no. 28), and a local committee of participating university hospitals individually approved the study. Because this is a retrospective study and the data analyzed were anonymized, informed consent from participants was obtained through an opt-out method on the web-site of each participating university hospitals in accordance with the Ethical Guidelines for Medical and Health Research Involving Human Subjects in Japan. The administrative office of J-CKD-DB project is in Kawasaki Medical School and is in charge of data cleansing and maintenance.

In the future, the J-CKD-DB will be linked to other databases. Firstly, linkage to J-RBR/J-KDR^7,8^, which has over 40,000 participants, is planned in late 2020 (Fig. 2). Therefore, to enhance future analysis, physicians in each university hospitals have manually flagged special registrations in the database as follows: (1) haemodialysis cases, (2) peritoneal dialysis cases, (3) kidney transplantation cases, (4) cases in which kidney biopsy is being performed, and (5) cases registered in the J-RBR.

### Participants

Inclusion criteria of J-CKD-DB are as follows: 1) Age ≥ 18 years old, 2) Proteinuria ≥ 1 + (dipstick test) according to the previous studies^4,5^ and/or estimated glomerular filtration rate (eGFR) <60 mL/min/1.73 m^2^ where estimated GFR is calculated using Japanese equation of eGFR: eGFR (mL/min/1.73 m^2^) = 194 × serum creatinine value ^−1.094^ × age^−0.287^ (female × 0.739)^22^. The registry includes all patients with CKD (both out- and inpatients) in the facilities participating in J-CKD-DB.

### Data collection

Baseline data abstraction and compilation were done from 1 January 2014 to 31 December 2014. To avoid input error and burden on physicians, all data elements are extracted automatically using SS-MIX2 storage and send to J-CKD-DB data centre (Fig. 1). Fundamental standards adopted in SS-MIX2 are Health Level Seven (HL7) V2.5 (ISO 27931)^23^ data format for patient profile, prescriptions (HOT reference code^24^), laboratory test results (Japan Laboratory Code Version 10 [JLAC10] code^25^), diagnoses (International Classifications of Disease, 10th revision [ICD10]^26^), incidences of major outcomes (such as death, hospital admission, discharge and transfer to other hospitals), etc.

CKD G staging was classified according to the Kidney Disease: Improving Global Outcomes (KDIGO) guideline^27^, with an eGFR ≥90, 60–89, 45–59, 30–44, 15–29, and <15 mL/min/1.73 m2 classified as G1, G2, G3a, G3b, G4, and G5, respectively. Dipstick proteinuria was classified into 3 categories: KDIGO A1 category of negative proteinuria (−); A2 category of trace proteinuria (±); and A3 category of ≥1 + ^3,28^. In the initial analysis, the patients with eGFR <5 ml/min/1.73m^2^ were excluded to eliminate the possibility of including patients with ESRD, because we could not distinguish those on hemodialysis, peritoneal dialysis, or those after kidney transplantation as of conducting the initial analysis. Furthermore, patients with eGFR ≥ 200 mL/min/1.73 m^2^ were excluded as clinically implausible^29^. Patients with eGFR ≥ 60 mL/min/1.73 m^2^ without trace proteinuria who did not meet the definition of CKD were excluded.

### Statistical analyses

Values are presented as median with interquartile interval (IQI), mean (SD), or count with percentage, as appropriate. Distributions of the variables were evaluated by histogram, quantile-quantile plot, and the Kolmogorov-Smirnov test. Clinical parameters were compared among the eGFR or proteinuria categories using the Kruskal–Wallis nonparametric test. All data were statistically analyzed using IBM SPSS Advanced Statistical Version 26.0 (SPSS, Chicago, IL, USA), and p < 0.05 was considered to indicate a significant difference.

## Supplementary information

Supplementary Information.

## Data Availability

The datasets used and/or analysed during the current study are not an open-access. However, we would encourage external investigators to consider applying to use the data for secondary analyses, to maximize the scientific output from the data. Further details on the J-CKD-DB, including information for joining and a full list of publications, are available from the website: http://j-ckd-db.jp/english/index.html.
