# Supplementary material for: J-CKD-DB: a nationwide multicentre electronic health record-based chronic kidney disease database in Japan
Source: Sci Rep. 2020 Apr 30;10:7351. doi: 10.1038/s41598-020-64123-z (PMC7192920; doi:10.1038/s41598-020-64123-z)

**Supplementary Information**

**J-CKD-DB: a nationwide multicentre electronic health record-based chronic kidney disease database in Japan**

Naoki Nakagawa^1^, Tadashi Sofue^2^, Eiichiro Kanda^3^, Hajime Nagasu^4^, Kunihiro Matsushita^5^, Masaomi Nangaku^6^, Shoichi Maruyama^7^, Takashi Wada^8^, Yoshio Terada^9^, Kunihiro Yamagata^10^, Ichiei Narita^11^, Motoko Yanagita^12^, Hitoshi Sugiyama^13^, Takashi Shigematsu^14^, Takafumi Ito^15^, Kouichi Tamura^16^, Yoshitaka Isaka^17^, Hirokazu Okada^18^, Kazuhiko Tsuruya^19,20^, Hitoshi Yokoyama^21^, Naoki Nakashima^22^, Hiromi Kataoka^23^, Kazuhiko Ohe^24^, Mihoko Okada^25^, Naoki Kashihara^4^

^1^Division of Cardiology, Nephrology, Respiratory and Neurology, Department of Internal Medicine, Asahikawa Medical University, Asahikawa, Japan; ^2^Division of Nephrology and Dialysis, Department of Cardiorenal and Cerebrovascular Medicine, Kagawa University, Kagawa, Japan; ^3^Medical Science, Kawasaki Medical School, Kurashiki, Japan; ^4^Department of Nephrology and Hypertension, Kawasaki Medical School, Kurashiki, Japan; ^5^Department of Epidemiology, Johns Hopkins Bloomberg School of Public Health, Baltimore, USA; ^6^Division of Nephrology and Endocrinology, University of Tokyo Graduate School of Medicine, Tokyo, Japan; ^7^Division of Nephrology, Nagoya University Graduate School of Medicine, Nagoya, Japan; ^8^Division of Nephrology, Department of Nephrology and Laboratory Medicine, Kanazawa University, Kanazawa, Japan; ^9^Department of Endocrinology, Metabolism and Nephrology, Kochi Medical School, Kochi University, Kochi, Japan; ^10^Department of Nephrology, Faculty of Medicine, University of Tsukuba, Tsukuba, Japan; ^11^Division of Clinical Nephrology and Rheumatology, Niigata University Graduate School of Medical and Dental Sciences, Niigata, Japan; ^12^Department of Nephrology, Graduate School of Medicine, Kyoto University, Kyoto, Japan; ^13^Department of Human Resource Development of Dialysis Therapy for Kidney Disease, Okayama University Graduate School of Medicine, Dentistry and Pharmaceutical Sciences, Okayama, Japan; ^14^Division of Nephrology, Department of Internal Medicine, Wakayama Medical University, Wakayama, Japan; ^15^Division of Nephrology, Faculty of Medicine, Shimane University, Izumo, Japan; ^16^Department of Medical Science and Cardiorenal Medicine, Yokohama City University Graduate School of Medicine, Yokohama, Japan; ^17^Department of Nephrology, Osaka University Graduate School of Medicine, Suita, Japan; ^18^Department of Nephrology, Faculty of Medicine, Saitama Medical University, Saitama, Japan; ^19^Department of Integrated Therapy for Chronic Kidney Disease, Kyushu University, Fukuoka, Japan; ^20^Department of Nephrology, Nara Medical University, Kashihara, Japan; ^21^Department of Nephrology, Kanazawa Medical University School of Medicine, Ishikawa, Japan; ^22^Department of Advanced Information Technology, Kyushu University, Fukuoka, Japan; ^23^Faculty of Health Science and Technology, Kawasaki University of Medical Welfare, Kurashiki, Japan; ^24^Department of Healthcare Information Management, The University of Tokyo Hospital, Tokyo, Japan; ^25^Institute of Health Data Infrastructure for All, Tokyo, Japan

Corresponding Author: Naoki Nakagawa, MD, PhD, Cardiology, Nephrology, Respiratory and Neurology, Department of Internal Medicine, Asahikawa Medical University, 2-1-1-1 Midorigaoka-higashi, Asahikawa, Japan

Phone: +81-166-68-2442, Fax: +81-166-68-2449

E-mail: [naka-nao@asahikawa-med.ac.jp](mailto:naka-nao@asahikawa-med.ac.jp)

**Supplementary Table S1.** Facilities participating in J-CKD-DB as of January 2018.

**Supplementary Table S2.** Participants' stratification according to eGFR category, and age, sex, and proteinuria strata.

**Supplementary Table S3.** Participants' stratification according to dipstick proteinuria category, and age and sex strata.

**Supplementary Figure 1.** Flow chart of CKD outpatients in this study.

**Supplementary Table S1.** Facilities participating in J-CKD-DB as of January 2018.

| Hospital name | Location of hospital (Prefecture) |
| --- | --- |
| Phase 1 Database-Building Hospitals | |
| Asahikawa Medical University | Hokkaido |
| University of Tsukuba | Ibaraki |
| University of Tokyo | Tokyo |
| Niigata University | Niigata |
| Kanazawa University | Kanazawa |
| Wakayama Medical University | Wakayama |
| Kawasaki Medical University | Okayama |
| Kagawa University | Kagawa |
| Kochi University | Kochi |
| Shimane University | Shimane |
| Kyushu University | Kyusyu |
| Phase 2 Database-Building Hospitals | |
| Jichi Medical University | Tochigi |
| Tokyo Women's Medical University | Tokyo |
| Teikyo University | Tokyo |
| Yokohama City University | Kanagawa |
| Nagoya University | Aichi |
| Fukui University | Fukui |
| Kyoto University | Kyoto |
| Osaka University | Osaka |
| Kobe University | Hyogo |
| Okayama University | Okayama |

**Supplementary Table S2.** Participants' stratification according to eGFR category, and age, sex, and proteinuria strata.

|  | eGFR category | | | | | |  | |
| --- | --- | --- | --- | --- | --- | --- | --- | --- |
|  | G1 | G2 | G3a | G3b | G4 | G5 | P value^a^ | |
| Mean eGFR (SD), mL/min/1.73 m^2^ | 108.4 | 72.7 | 53.6 | 39.0 | 23.9 | 9.1 | <0.001 | |
|  | (18.4) | (8.3) | (4.2) | (4.2) | (4.2) | (3.1) |  | |
| Cases | 1,001 | 2,612 | 23,333 | 8,357 | 2,710 | 1,108 |  | |
| Prevalence | 2.6% | 6.7% | 59.6% | 21.4% | 6.9% | 2.8% |  | |
| Dipstick proteinuria monitoring | 1,001 | 2,612 | 9,276 | 4,008 | 1,664 | 494 | <0.001 | |
|  | (100%)* | (100%)* | (39.8%)* | (48.0%)* | (61.4%)* | (44.6%)* |  | |
| Stratified prevalence |  |  |  |  |  |  |  | |
| Age strata |  |  |  |  |  |  |  | |
| 18–44 years | 610 | 641 | 718 | 223 | 131 | 101 | <0.001 | |
|  | (25.2%) | (26.4%) | (29.6%) | (9.2%) | (5.4%) | (4.2%) |  |  |
| 45–64 years | 252 | 948 | 5,815 | 1,330 | 489 | 332 |  |  |
|  | (2.7%) | (10.3%) | (63.4%) | (14.5%) | (5.3%) | (3.6%) |  |  |
| 65–74 years | 85 | 590 | 8,146 | 2,472 | 688 | 322 |  |  |
|  | (0.7%) | (4.8%) | (66.2%) | (20.1%) | (5.6%) | (2.6%) |  |  |
| 75–84 years | 44 | 342 | 6,994 | 3,131 | 908 | 276 |  |  |
|  | (0.4%) | (2.9%) | (59.8%) | (26.8%) | (7.8%) | (2.4%) |  |  |
| 85+ years | 10 | 91 | 1,660 | 1,201 | 494 | 77 |  |  |
|  | (0.3%) | (2.6%) | (47.0%) | (34.0%) | (14.0%) | (2.2%) |  |  |
| Sex strata |  |  |  |  |  |  |  | |
| Men | 444 | 1,606 | 12,661 | 4,614 | 1,434 | 651 | <0.001 | |
|  | (2.1%) | (7.5%) | (59.1%) | (21.6%) | (6.7%) | (3.0%) |  |  |
| Women | 557 | 1,006 | 10,672 | 3,743 | 1,276 | 457 |  |  |
|  | (3.1%) | (5.7%) | (60.3%) | (21.1%) | (7.2%) | (2.6%) |  |  |
| Dipstick proteinuria strata |  |  |  |  |  |  |  | |
| (-) | 0 | 0 | 6,652 | 2,133 | 533 | 39 | <0.001 | |
|  | (0.0%) | (0.0%) | (71.1%) | (22.8%) | (5.7%) | (0.4%) |  |  |
| (±) | 227 | 604 | 1,302 | 693 | 257 | 43 |  |  |
|  | (7.3%) | (19.3%) | (41.7%) | (22.2%) | (8.2%) | (1.4%) |  |  |
| (1+) | 557 | 1377 | 778 | 592 | 358 | 121 |  |  |
|  | (14.7%) | (36.4%) | (20.6%) | (15.6%) | (9.5%) | (3.2%) |  |  |
| (2+) | 150 | 492 | 409 | 401 | 309 | 158 |  |  |
|  | (7.8%) | (25.6%) | (21.3%) | (20.9%) | (16.1%) | (8.2%) |  |  |
| (3+) | 65 | 121 | 121 | 164 | 189 | 124 |  |  |
|  | (8.3%) | (15.4%) | (15.4%) | (20.9%) | (24.1%) | (15.8%) |  |  |
| (4+) | 2 | 18 | 14 | 25 | 18 | 9 |  |  |
|  | (2.3%) | (20.9%) | (16.3%) | (29.1%) | (20.9%) | (10.5%) |  |  |

# *Prevalence of each eGFR category; ^a^Kruskal–Wallis test for difference across groups.

# Supplementary Table S3. Participants' stratification according to dipstick proteinuria category, and age and sex strata.

|  | Proteinuria category | | | | | |  |
| --- | --- | --- | --- | --- | --- | --- | --- |
|  | (-) | (±) | (1+) | (2+) | (3+) | (4+) | P value^a^ |
| Cases | 9,357 | 3,126 | 3,783 | 1,919 | 784 | 86 |  |
| Prevalence | 49.1% | 16.4% | 19.9% | 10.1% | 4.1% | 0.5% |  |
| Stratified prevalence |  |  |  |  |  |  |  |
| Age strata |  |  |  |  |  |  |  |
| 18–44 years | 291 | 367 | 797 | 329 | 152 | 24 | <0.001 |
|  | (14.8%) | (18.7%) | (40.7%) | (16.8%) | (7.8%) | (1.2%) |  |
| 45–64 years | 2,315 | 784 | 1,120 | 614 | 239 | 32 |  |
|  | (45.4%) | (15.4%) | (21.9%) | (12.0%) | (4.7%) | (0.6%) |  |
| 65–74 years | 3,342 | 897 | 913 | 486 | 198 | 19 |  |
|  | (57.1%) | (15.3%) | (15.6%) | (8.3%) | (3.4%) | (0.3%) |  |
| 75–84 years | 2,775 | 814 | 713 | 364 | 159 | 7 |  |
|  | (57.4%) | (16.8%) | (14.8%) | (7.5%) | (3.3%) | (0.1%) |  |
| 85+ years | 634 | 264 | 240 | 126 | 36 | 4 |  |
|  | (48.6%) | (20.2%) | (18.4%) | (9.7%) | (2.8%) | (0.3%) |  |
| Sex strata |  |  |  |  |  |  |  |
| Men | 4,868 | 1,767 | 2,200 | 1,240 | 533 | 66 | <0.001 |
|  | (45.6%) | (16.6%) | (20.6%) | (11.6%) | (5.0%) | (0.6%) |  |
| Women | 4,489 | 1,359 | 1,583 | 679 | 251 | 20 |  |
|  | (53.6%) | (16.2%) | (18.9%) | (8.1%) | (3.0%) | (0.2%) |  |

^a^Kruskal–Wallis test for difference across groups.

**Supplementary Figure S1.** Flowchart of CKD outpatients in this study.


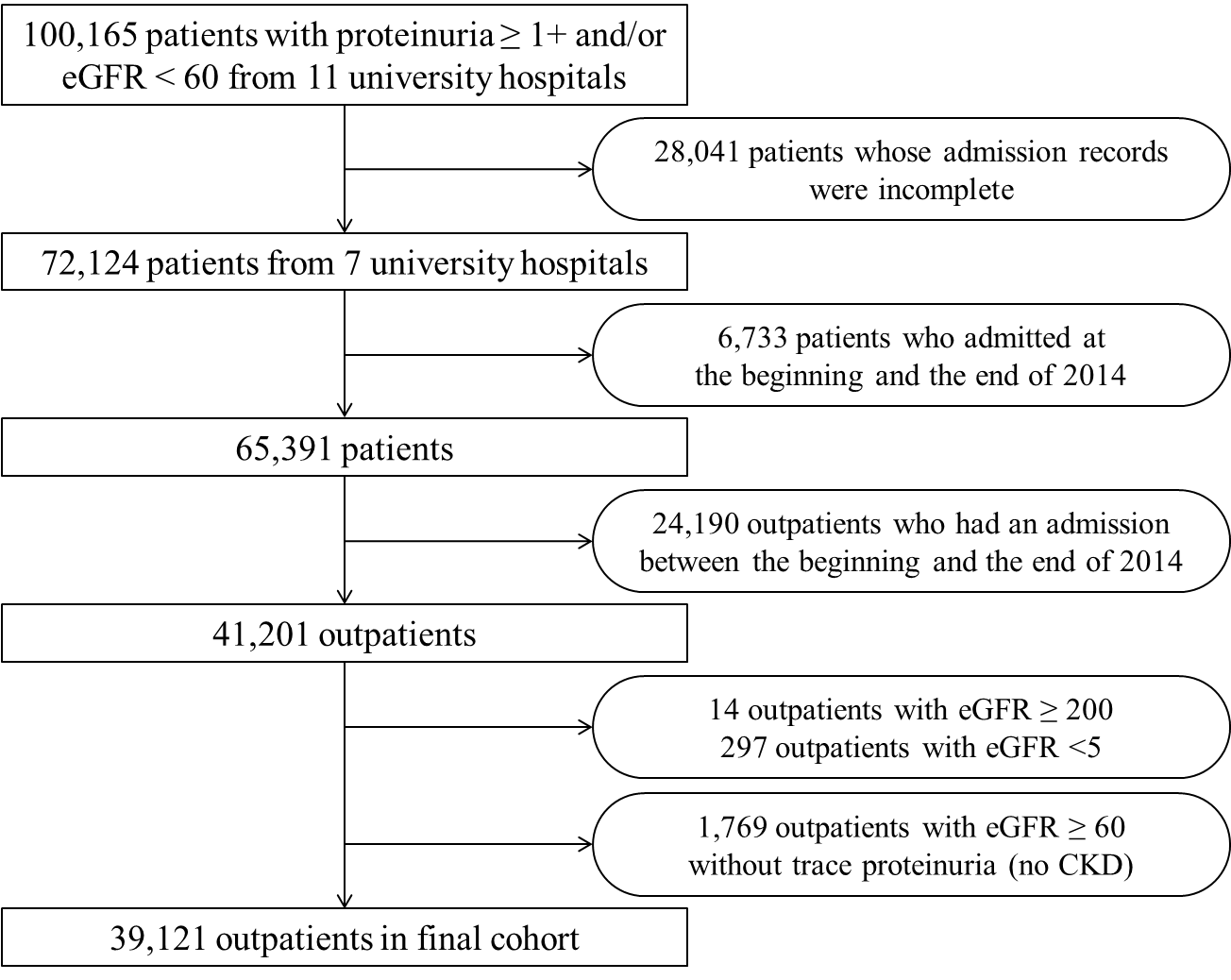

Supplement: Supplementary file 1 — Supplementary Information. [file 41598_2020_64123_MOESM1_ESM.docx]
